# Regulatory network analysis based on integrated miRNA-TF reveals key genes in heart failure

**DOI:** 10.1038/s41598-024-64732-y

**Published:** 2024-06-17

**Authors:** Ziyue Zhang, Ziying Zou, Hui Zhang, Dai-Min Zhang

**Affiliations:** Department of Cardiology, Sir Run Run Hospital, Nanjing Medical University, 109 Longmian Road, Nanjing, 211112 Jiangsu People’s Republic of China

**Keywords:** Heart failure, Methylation, Transcription factor, Comprehensive analysis, microRNAs, Cardiology, Diseases

## Abstract

The etiology and pathophysiology of heart failure are still unknown. Increasing evidence suggests that abnormal microRNAs (miRNAs) and transcription factors (TFs) expression may be associated with the development of heart failure. Therefore, this study aims to explore key miRNAs, TFs, and related genes in heart failure to gain a greater understanding of the pathogenesis of heart failure. To search and download the dataset of mRNA chips related to heart failure from the GEO database (GSE59867, GSE9128, and GSE134766), we analyzed differential genes and screened the common differentially expressed genes on two chips using R language software. The binary interactions and circuits among miRNAs, TFs, and corresponding genes were determined by Pearson correlation coefficient. A regulatory network of miRNAs, TFs, and target genes was constructed based on bioinformatics. By comparing the sequences of patients with and without heart failure, five downregulated genes with hypermethylated mRNA and three upregulated genes with hypomethylated mRNA were identified. The miRNA-TF gene regulatory network consisted of 26 miRNAs, 22 TFs and six genes. GO and KEGG analysis results revealed that BP terms like cellular response to organic substance, cellular response to cytokine stimulus, and KEGG pathways like osteoclast differentiation, MAPK signaling pathway, and legionellosis were enriched of the DEGs. *TMEM87A*, *PPP2R2A*, *DUSP1*, and miR-92a have great potential as biomarkers for heart failure. The integrated analysis of the mRNA expression spectrum and microRNA-transcription factor-gene revealed the regulatory network of heart failure, which may provide clues to its alternative treatment.

## Introduction

Heart failure (HF) is a group of clinical syndromes caused by various cardiac structural or functional diseases, leading to absolute or relative decreases in cardiac output^1^. Epidemiologic data suggest that the incidence of heart failure in developed countries is approximately 1–2% of the adult population. There are currently 22.5 million cases of heart failure worldwide, and the number of heart failure cases is increasing by two million every year^2^. Despite tremendous developments in medical technology, heart failure, as the final stage of most cardiovascular diseases, is the only cardiovascular disease whose incidence, morbidity, and mortality rates are increasing every year.

Studies on silenced small RNAs have mainly followed two directions: small interfering RNAs (siRNAs) and microRNAs (miRNAs). siRNAs are mainly involved in RNA interference with target mRNA, while miRNAs are more involved in regulating gene expression and play greater roles in this capacity. siRNA, which is processed by the specific endonuclease dicer, allows for the selective reduction of specific protein production via RNA interference silencing complex^3^. Mechanically, siRNA binds to multienzyme complexes of RNA-induced silencing complexes and then localizes to specific sites on mRNA to degrade target mRNA through endonuclease and exonuclease activity^4^. For example, in diastolic heart failure (DHF) rat models, silencing the SOCS3 gene by administration of adeno-associated virus 9-mediated RNA interference targeting SOCS3 (AAV9-SOCS3 siRNA) significantly reduced myocardial fibrosis and inflammatory responses and improved cardiac function^5^. Therefore, RNA interference (RNAi) has great promise in the treatment of heart failure. miRNA is a compound noncoding RNA molecule with a length of 22–25 nt and is widely expressed in various cell tissues. miRNAs bind mRNA sequences in the 3′UTR, and also in the 5′UTR and CDS^6^, resulting in the inhibition of protein translation or gene expression at the post-transcriptional level, thereby precisely regulating cell growth, differentiation, and apoptosis^7,8^. Lu et al. found that miR-328 is involved in the remodeling of atrial fibrosis by regulating target genes of Ca^2+^ channels during the reconstruction of ion channels in atrial fibrillation^9^. In addition, disease-related miRNAs can be used as the entry point to determine genes and transcription factors (TFs) that interact with miRNAs through the principle of biological genetic information to construct a mixed coregulatory network of corresponding diseases^10^.

TFs are key regulators of gene expression that enhance or inhibit gene transcription by binding to specific DNA sequences of target gene promoters. If TFs and miRNAs jointly regulate a coding gene, and transcription factors in turn regulate the coding gene of this miRNA, a feed-forward loop (FFL) is established between transcription factors, miRNAs, and the coding gene^11^. Alternatively, when the TFs regulate the gene transcription of a miRNA and the mature miRNA inhibits the translation of the TFs at the posttranscriptional level, a regulatory feedback loop (FBL) is established. It has been suggested that hundreds of coregulatory modules may be composed of miRNAs and transcription factors in mammals. In addition, multiple feed-forward and feedback functional loops have been experimentally confirmed, including the feedback loop between transcription factors *PITX3* and miR-133b in the development of middle brain neurons^12^, the feedback loop formed by cell cycle genes *CCND1* and miR-17/20 in breast cancer^13^, and the feed-forward loop of TP53/miR-106b/E2F related to cell proliferation function^14^. Given the ubiquity of miRNA and TF coregulation and the important role of mRNA and TFs in complex diseases, we proposed a coregulation network of miRNA and TFs affecting gene expression to study heart failure.

In this project, we used the coregulation of miRNAs and TFs as the entry point, developed and applied bioinformatics methods, integrated experimental and predictive data, comprehensively and accurately constructed the coregulation FFL module formed between miRNAs, TFs, and heart failure genes, and established a coregulation miRNA-TF network. Through the statistical analysis of the regulatory network, miRNA and genes that play core roles in heart failure were identified. This is the first time that the pathogenesis and process of heart failure have been studied at the system level, which provides a theoretical basis for heart failure diagnosis and treatment and new ideas and methods for researching complex diseases.

## Methods

### Data sources

**Figure 1:**
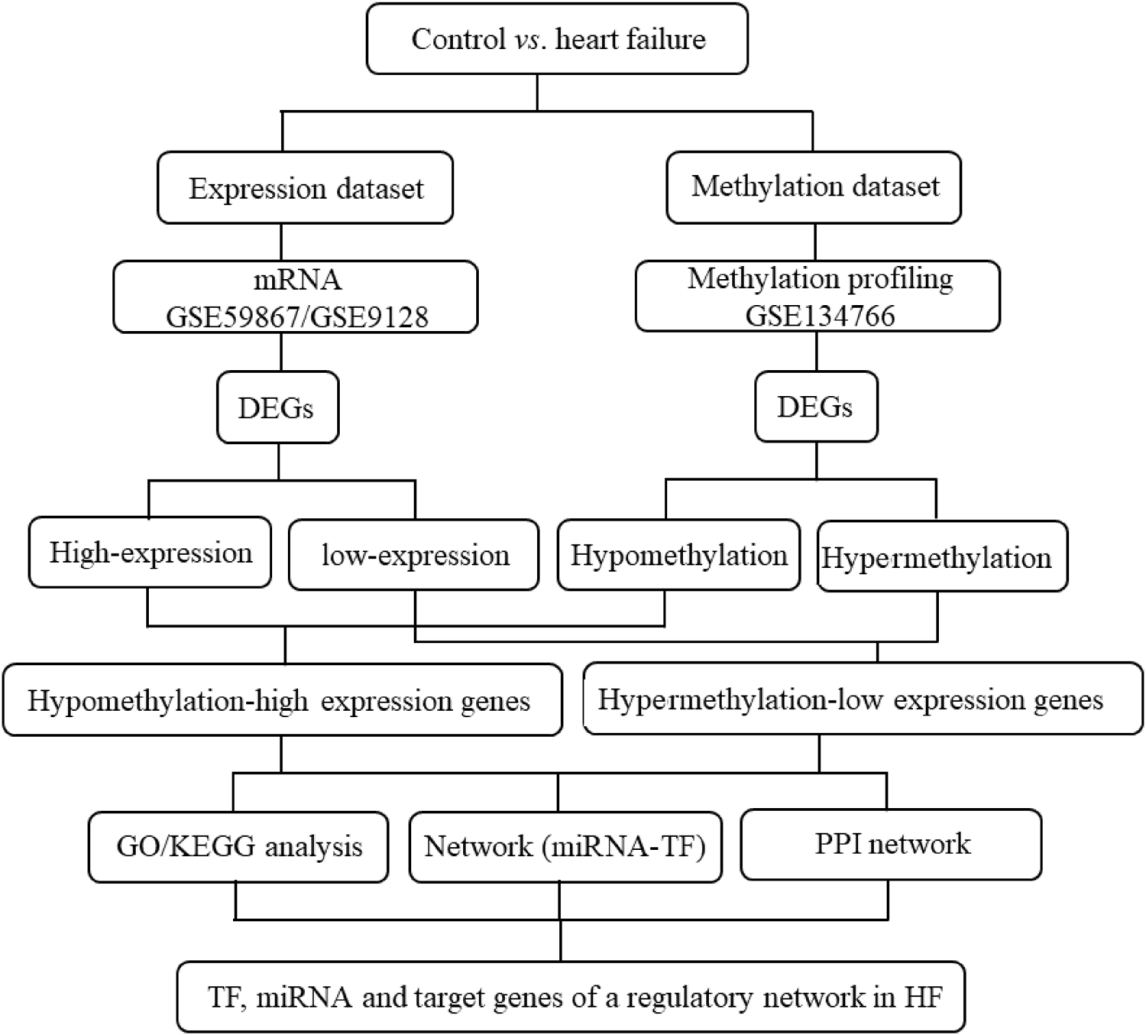
Flow chart of data preparation, processing, and analysis.

Gene Expression Omnibus (GEO, http://www.ncbi.nlm.nih.gov/geo/) database was utilized to download the expression and methylation data with HF (Fig. 1). Nine ST-segment elevation myocardial infarction-induced HF samples and eight healthy control samples were downloaded from the mRNA datasets GSE59867^15^. The other mRNA dataset GSE9128 contained three healthy and seven ischemic cardiomyopathy-induced HF samples that were analyzed in the study^16^. Furthermore, a methylation profiling by high throughput sequencing which contained 10 HF samples and 10 healthy samples was downloaded^17^.

### Data pre-processing

The mRNA datasets for HF analysis were extracted from the Series Matrix File, respectively. The probe ID was converted into a gene symbol or miRNA ID through the platform annotation information table. The same gene symbol or miRNA ID was incorporated. Expression matrixes of mRNA and miRNA samples were screened out according to ID. Other data sets were preprocessed in the same way. Then, NormalizeBetweenArrays in the limma package was applied to perform quartile standardization on the obtained chip expression data^18^. Then log2 logarithm conversion was performed to obtain the gene expression matrix of the sample group.

### Screening of differentially expressed genes (DEGs)

The empirical Bayes method in limma package was used to screen DEGs with the screening threshold as the corrected *P*-value < 0.05 and | logFC |≥ 1. After that, the heat maps reflecting the overall distribution trend of differential genes were drawn by the edger package^19^. Next, genes with upregulated or downregulated expression in the two data sets were intersected, respectively, using the online tool VENNY 2.1, and genes that showed both upregulated and downregulated expression were selected for further analysis.

### Sample correlation analysis

Principal component analysis (PCA) is an unsupervised feature learning data dimensionality reduction algorithm that shows the classification of data through the expression data of samples. PCA analysis can be used to obtain the intuitive distribution of samples between experimental groups and control groups, to facilitate the detection and removal of outlier samples, and to find sample sets with high similarity. A PCA analysis was performed on genes with a significant mean difference in expression in all samples (*p* ≤ 0.05), and a PCA diagram was generated.

The correlation of gene expression levels between samples is an important reference index for the reliability of experiments and the reasonable selection of samples. The correlation coefficient can represent similarity between samples. The higher the correlation coefficient is, the greater the similarity between the two samples is. The correlation coefficient between two samples was calculated using gene expression data, and a heat map was generated.

### TF-miRNA-mRNA regulatory network

By predicting the binding relationships between miRNAs and TFs using TransmiR^20^, miRNAs and mRNAs using miRMap^21^, miRanda^22^, miRDB^23^, TargetScan^21^ and miTarBase^24^, and mRNA and TF using ENCODE^25^, Cytoscape V3.7^26^ was used to construct the TF-miRNA-mRNA network.

### Protein interaction network analysis

STRING 10.5 (https://string-db.org)^27^ was used to construct a protein–protein interaction (PPI) network, and the lowest interaction score was set as 0.7. The analysis results were imported into cytoscape software for visualization.

### Gene function enrichment analysis

Based on the gene ontology database (https://www.geneontology.org) and the KEGG database (http://www.kegg.jp/ or http://www.genome.jp/kegg/) of biochemical pathways, the functional enrichment of candidate genes was analyzed. Fisher's exact test was used to determine the specific functional items that have the greatest correlation with a group of genes. In the analysis results, each item corresponds to a statistical p-value to represent the significance. The smaller the p-value is, the greater the correlation between this item and the input genes is.

## Results

### Common screened differential expressed genes (DEGs)

**Figure 2:**
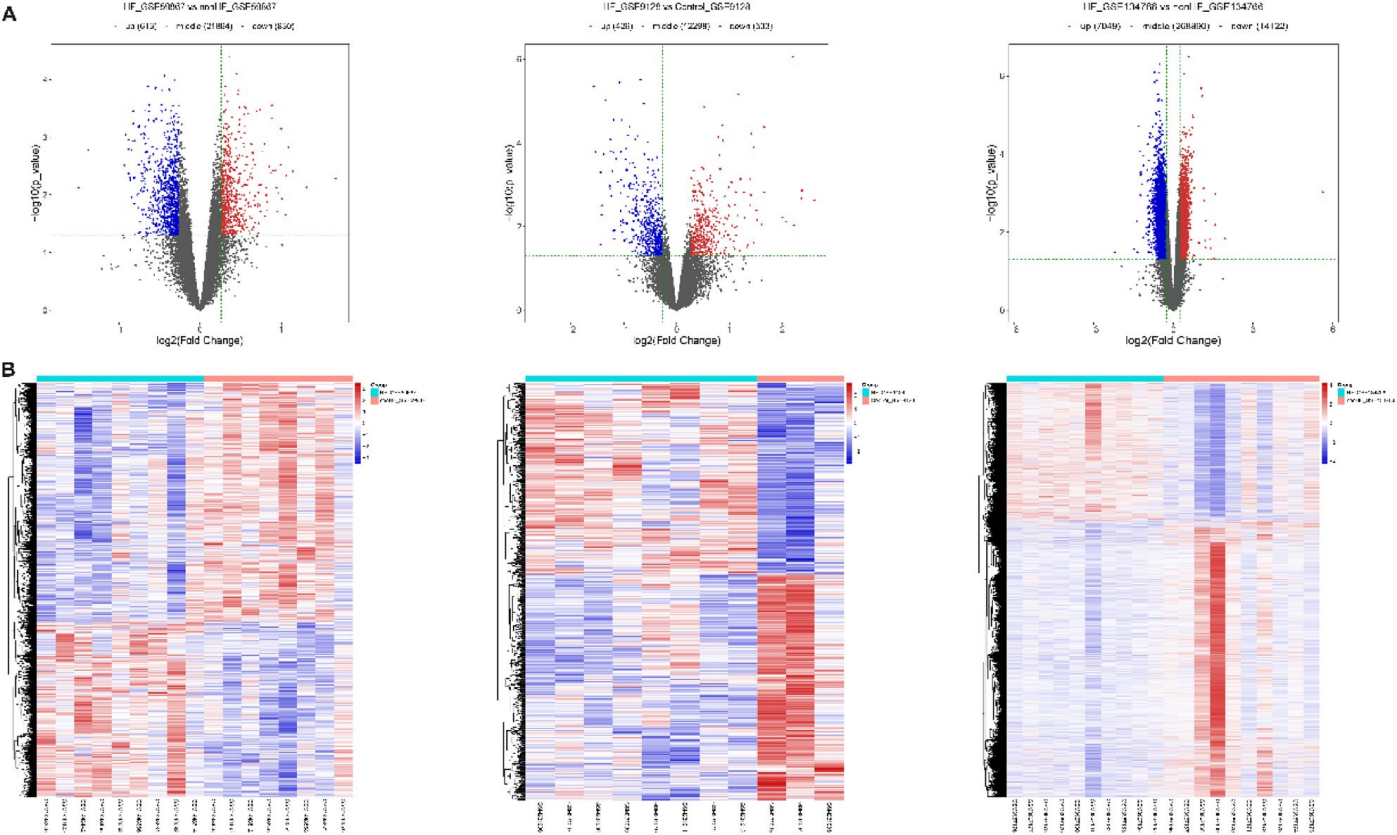
mRNA-seq, methylation-seq analysis and DEG profiling. (**A**) DEG profiling related volcano map based on the GSE59867, GSE9128 and GSE134766 datasets. (**B**) DEG profiling-related heat map based on the GSE59867, GSE9128, and GSE134766 datasets. The list shows the analyzed samples and the relative expression description is based on the color scale. Red indicates upregulation, and blue indicates downregulation.

Upon GSE59867 dataset screening, 613 upregulated and 830 downregulated differential genes were obtained from the left ventricular tissues of patients with heart failure; upon GSE9128 dataset screening, 426 upregulated and 503 downregulated differential genes were obtained from the left ventricular tissues of hypertensive patients with heart failure (*p* < 0.05, |logFC|≥ 1.2) as shown in Table 1. Gene expression is regulated by epigenetic mechanisms as well as gene–gene interactions. Therefore, through data mining of a comprehensive database, we screened HF-related DNA methylation and other differential epigenetic factors and conducted correlation analysis with differentially expressed genes to explore the regulatory relationships of epigenetic effects on gene expression. Finally, a total of 7049 hypermethylation and 14,122 hypomethylation sites were obtained in GSE134766 data sets as shown in Table 1. After that, 3945 corresponding genes with hypermethylation sites and 6150 corresponding genes with hypomethylation sites were confirmed, respectively. Volcanic and thermal maps of the differential genes are shown in Fig. 2A and B.

**Table 1:** Statistical overview of differential genes.

Data information		Difference threshold		Number of differential genes		Number of samples	
Data sources	Platform	FC	*P*_value	Up	Down	HF	Control
GSE59867	GPL6244	1.2	0.05	613	830	9	8
GSE9128	GPL96	1.2	0.05	426	503	8	3
GSE134766	GPL11154	1.2	0.05	7049	14122	10	10

**Figure 3:**
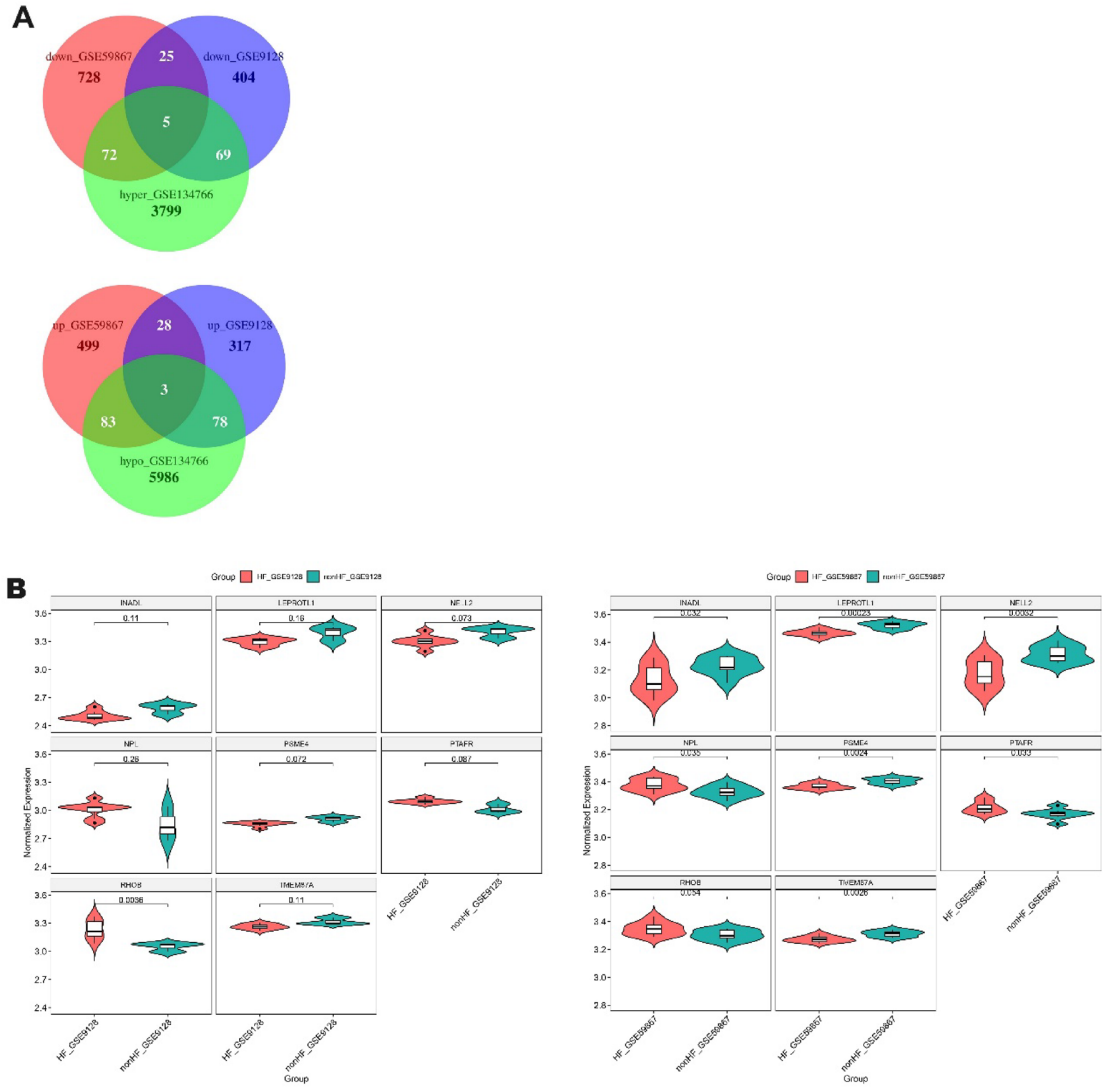
Methylation-seq analysis and DEG profiling. (**A**) Venn map of intersected hypermethylated genes and downregulated mRNA genes and Venn map of intersected hypomethylated genes and upregulated mRNA genes. Five hypermethylated downregulated mRNA genes, including *INADL, LEPROTL1, NELL2, PSME4* and *TMEM87A*, and three hypomethylated upregulated mRNAs, including *NPL*, *PTAFR* and *RHOB*. (**B**) Expression levels of the eight differentially expressed genes in heart failure and nonheart failure groups from the GSE59867 and GSE9128 datasets.

The differentially expressed genes that were upregulated and downregulated genes in the GSE59867 and GSE9128 datasets were intersected (Table 2), and ultimately, 31 differentially expressed genes that were upregulated and 30 differentially expressed genes that were downregulated were obtained (Fig. 3A, B). Here, the intersection of the differently expressed genes and the corresponding genes with the differently modified methylation sites were selected; namely, five downregulated hypermethylated mRNA genes, including *INADL*, *LEPROTL1*, *NELL2*, *PSME4* and *TMEM87A*, and three hypomethylated upregulated mRNAs, including *NPL*, *PTAFR* and *RHOB*, were used for subsequent analysis (Fig. 3A). The specific expression levels of these eight differentially expressed genes from the GSE59867 and GSE9128 datasets in the heart failure and nonheart failure groups are shown in Fig. 3B.

**Table 2:** Intersection of differential genes between GSE59867 and GSE9128.

Up-regulated differentially expressed genes	Down-regulated differentially expressed genes
AP2M1, AQP9, BCL6, BEST1, CEBPD, CSF2RA, CXCL2, DUSP1, FOS, FPR2, G0S2, GLUL, HSPA1A, IL1B, ILK, LAT2, LILRA2, LILRB2, NAMPT, NFIL3, NPL, POLR2E, PSME3, PTAFR, RHOB, RNASE6, RNF5, STX11, TLR5, TREM1, TRIB1	ALG9, AP3M2, CAPRIN2, CYLD, EBLN2, FAM153A, GPRASP1, HIVEP1, INADL, KDM3A, KLHDC2, LEPROTL1, LRRC16A, MCM6, MGAT4A, MS4A3, MTR, NELL2, OFD1, PCNXL2, PPP2R2A, PPP3CC, PSME4, RGCC, SATB1, SFXN1, TAF1D, TMEM87A, ZBTB25, ZC3HAV1

### Enrichment analysis of the GO functions of differentially expressed genes

According to the selected differentially methylated and differentially expressed intersected genes, GO and KEGG enrichment analyses were performed. The functions of genes can be divided into three categories: biological process (BP), molecular function (MF) and cellular component (CC).

**Figure 4:**
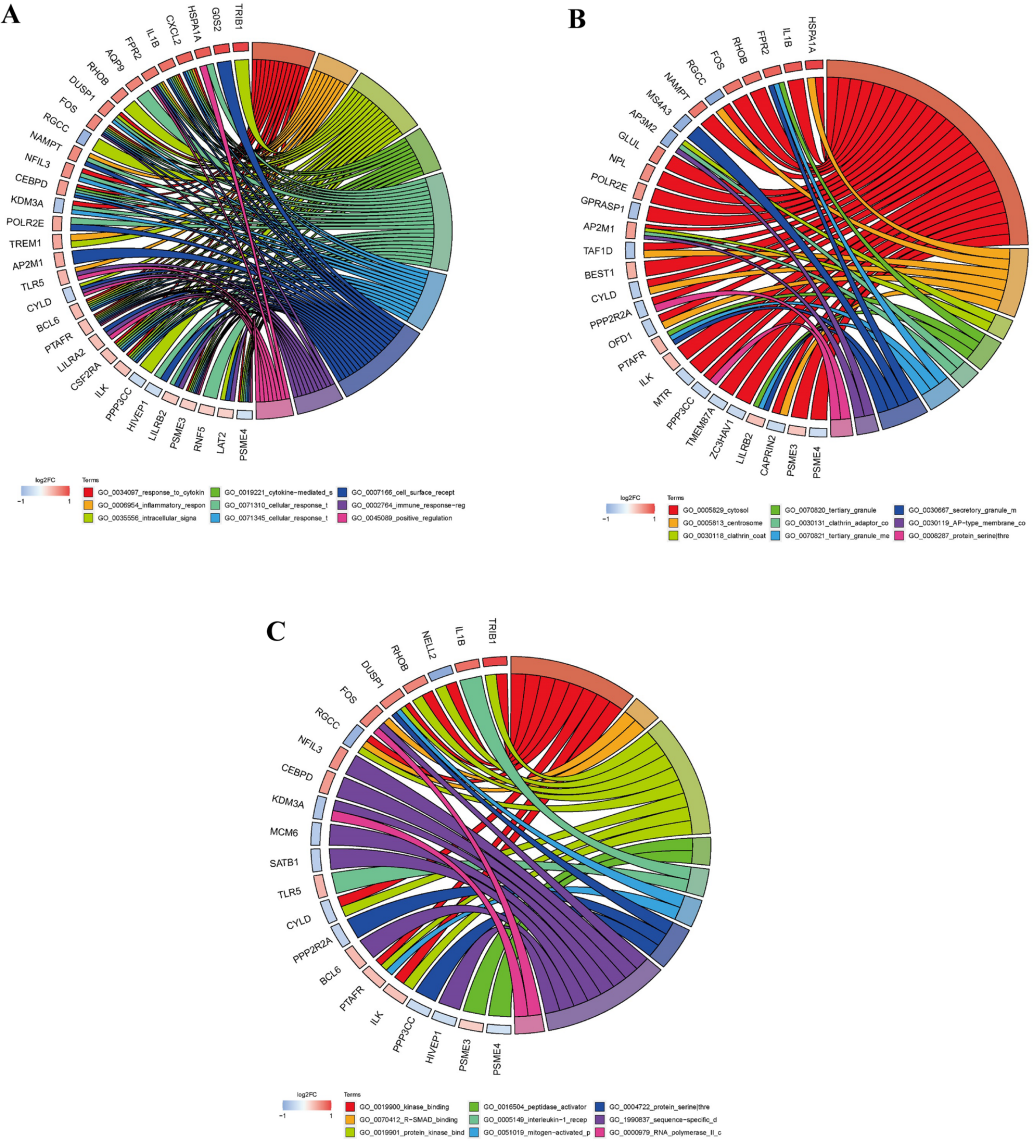
Enrichment analysis of GO functions of the differentially expressed genes. GO terms rich in differentially expressed genes involved in regulatory networks. (**A**) BP represents biological process. (**B**) CC represents cellular component. (**C**) MF represents molecular function.

**Figure 5:**
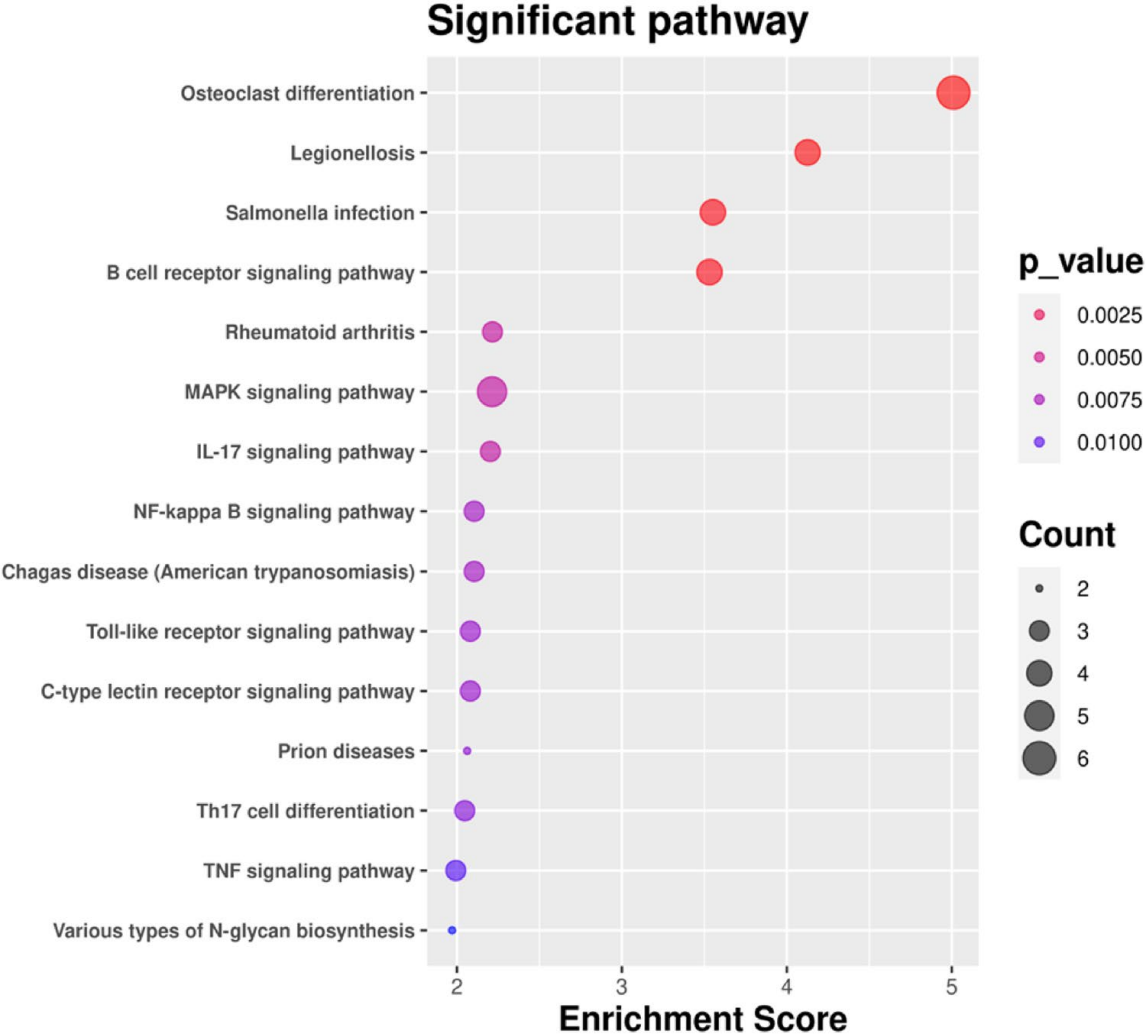
Enrichment analysis of differentially expressed genes in KEGG pathways. Osteoclast differentiation, MAPK signaling pathway, legionellosis, bacterial infection and the B-cell receptor signaling pathway accounted for the majority of the KEGG pathways.

The results showed that in the biological process category of GO terms (GO-BP), the common differential genes in heart failure patients were mainly concentrated in cellular response to organic substance, cellular response to cytokine stimulus, positive regulation of innate immune response, immune response-regulating signaling pathway, and inhibitory response (Fig. 4A). Target genes in the GO cell component category (GO-CC) were enriched in tertiary granule membrane, clathrin adaptor complex, and AP-type membrane coat adaptor complex, and clathrin coat as well as protein serine threonine phosphatase complex (Fig. 4B). The GO molecular function (GO-MF) category was enriched in genes involved in protein kinase binding, interleukin-1 receptor binding, protein serine threonine phosphatase activity, kinase binding and R-SMAD binding (Fig. 4C). The KEGG results showed that osteoclast differentiation, MAPK signaling pathway, legionellosis, bacterial infection and B cell receptor signaling pathway accounted for the majority of the enriched genes (Fig. 5).

### Protein interaction network analysis

**Figure 6:**
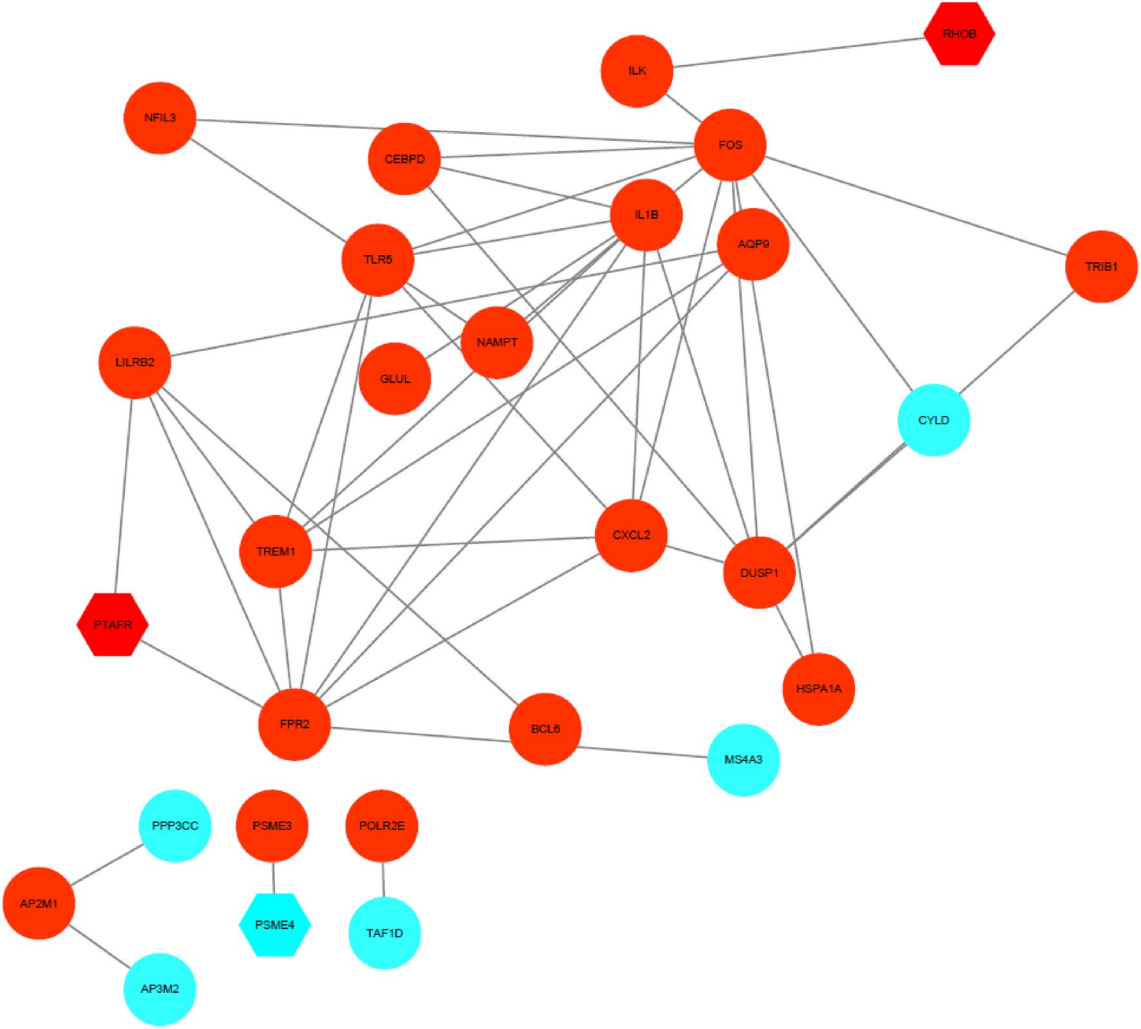
PPI network constructed with hub genes. The blue circles indicate downregulated genes, and the red circles indicate upregulated genes.

To further explore the interactions between HF-related differentially expressed genes and other genes, the protein interactions of each gene were first analyzed individually using STRING, and the interaction records of the proteins encoded by each gene were extracted and preserved with the intermediate reliability (0.40) as the lowest interaction score. The results showed that hub genes included *FOS*, *FPR2*, *IL1B*, *DUSP1* and *TLR5* (Fig. 6). The lack of relevant studies resulted in an insufficient number of annotations; however, the small number of interacting proteins does not indicate that a protein encoded by a gene has a weak regulatory effect. After in-depth observation, we found that some factors have been shown to play important roles in the pathogenesis of HF, such as *RHOB* and *TMEM87A*.

### The TF-miRNA-mRNA regulatory network

**Figure 7:**
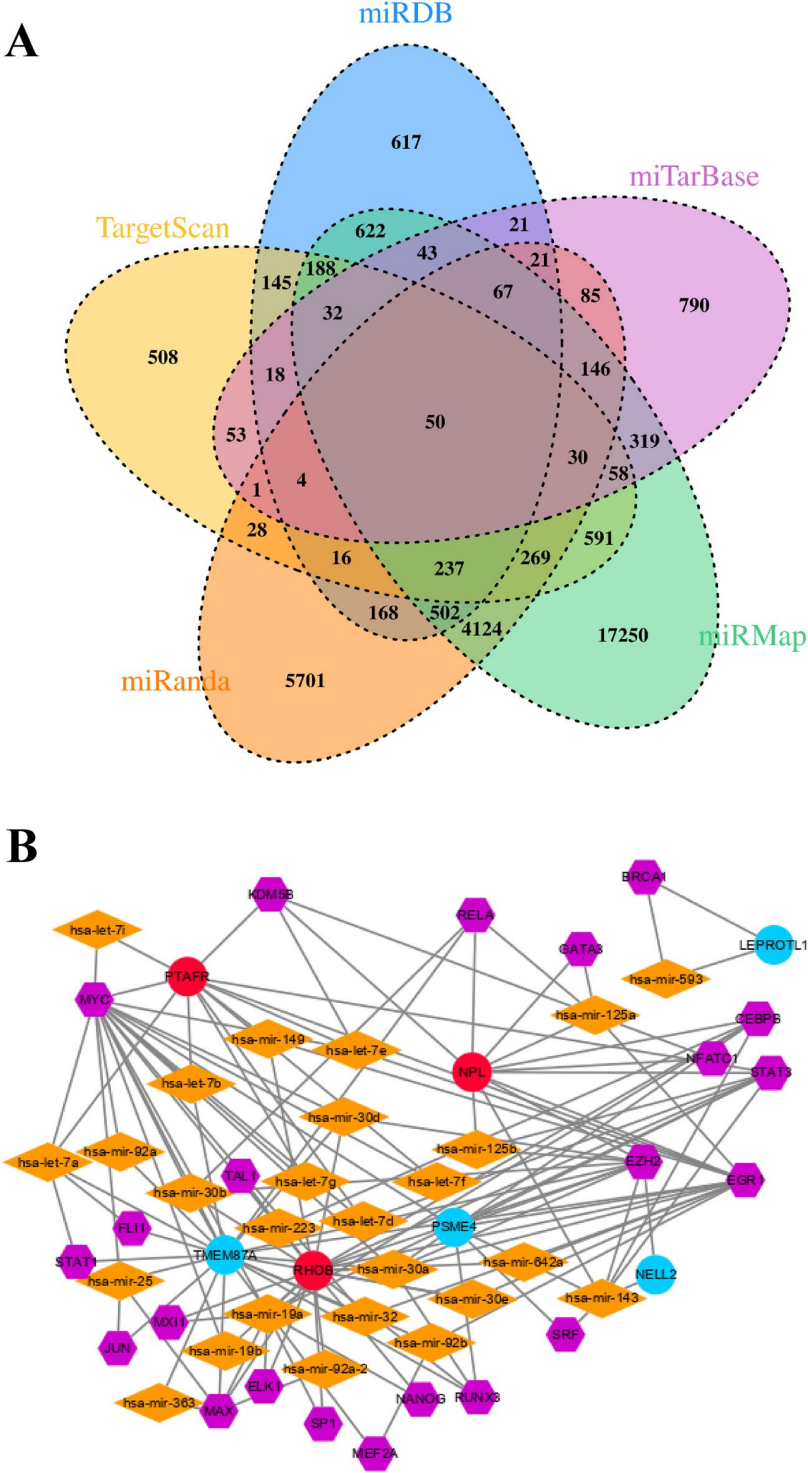
The TF-miRNA-mRNA regulatory network. (**A**) Intersection of miRNA-target genes relational pairs from the database. (**B**) Integrated regulatory network of miRNA-transcription factor-genes related to heart failure. Diamonds represent miRNAs, circles represent genes, and hexagons represent TFs. Red circles represent upregulated genes, and blue circles represent down-regulated genes.

According to the miRMap, miRanda, miRDB, TargetScan and miRTarBase databases, the miRNA corresponding to the abovementioned differential methylation and differential expression intersection genes (at least for the related pairs existing in three databases) were found. A total of 259 pairs of miRNAs and genes were extracted for subsequent construction of a TF-miRNA-mRNA regulatory network. According to the data on the level of TF miRNA regulation data based on different categories in the TransmiR v2.0 database (http://www.cuilab.cn/transmir), 780 pairs of miRNAs and TFs were found. Then, the genes in these miRNA-gene relationship pairs were identified, and the corresponding gene-TF relationship was identified according to the ENCODE database for a total of 709 pairs. Based on the relationship between binary interactions and circuits, we constructed the miRNA-TF gene regulatory network (Fig. 7). The final integrated network contained 26 miRNAs ( hsa-mir-143, hsa-mir-25, hsa-mir-30a, hsa-mir-30b, hsa-mir-30d, hsa-mir-30e, hsa-mir-32, hsa-mir-363, hsa-mir-593, hsa-mir-92a, hsa-mir-92b, hsa-let-7a, hsa-let-7b, hsa-let-7d, hsa-let-7e, hsa-let-7f., hsa-let-7g, hsa-let-7i, hsa-mir-125a, hsa-mir-125b, hsa-mir-149, hsa-mir-19a, hsa-mir-19b, hsa-mir-223, hsa-mir-92a-2, hsa-mir-642a ), 22 TFs ( *EZH2, MAX, MYC, JUN, EGR1, RUNX3, RELA, BRCA1, MEF2A, STAT3, EGR1, KDM5B, GATA3, KDM5B, NFATC1, CEBPB, ELK1, MXI1, SP1, NANOG, TAL1, RUNX3*) and six genes ( *NELL2, TMEM87A, LEPROTL1, PTAFR, NPL, RHOB* ).The top five key regulators in the miRNA-TF gene network in heart failure were *TMEM87A*, *RHOB*, *PTAFR*, *MYC* and *NPL*, which were directly related to 22, 21, 19, 12 and 11 corresponding targets, respectively (Table 1).

## Discussion

In this study, we used the expression and methylation data about HF to identify the mRNAs, miRNAs and TFs involved in transcriptome alteration. By intersecting different data sets, five down-regulated and three up-regulated genes were obtained, including *INADL*, *LEPROTL1*, NELL2, *PSME4* and *TMEM87A*, *NPL*, *PTAFR* and *RHOB*. Finally, a PPI network and a comprehensive network combined a TF-miRNA-mRNA network which contained 26 miRNAs, 22 TFs, and six target genes were build.

The miRNA-TF-target gene feed-forward loop refers to transcription factors upstream of miRNAs that not only indirectly regulate target genes through miRNAs but that also directly regulate target genes; that is, a miRNA and its upstream transcription factor jointly regulate the same target gene. As the results, Transmembrane protein 87A (*TMEM87A*) was the only marker which was found in the interaction among the three datasets and the TF-miRNA-mRNA network. *TMEM87A* has been proven to modulate melanoma adhesion and migration as a component of a novel mechanoelectrical transduction pathway^28^. In addition, *TMEM87A* could promote metastasis of gastric cancer and cell proliferation by elevating ULK1 via sponging miR-142-5p^29^. No direct evidence was associated *TMEM87A* with HF in the previous researches. However, twenty-one genes including *TMEM87A* were revealed overexpressed in both hypertensive and left ventricular remodeling^30^. By integrated bioinformatic analyses, *TMEM87A* was also identified as target genes and transcription factors in mice with dilated cardiomyopathy. In future work, more data and work are needed to verify the correlation between *TMEM87A* and HF. Among the TF-miRNA-mRNA network, special AT-rich sequence-binding protein 1 (SATB1) is important for chromatin, and is involved in the regulation of hundreds of genes^31^. Recent integration of HF has revealed an important feature of protein phosphatase 2A (PP2A, encoded by the *PPP2R2A* gene) by regulating Ca^2+^ transient in failing heart^32^. Based on their unique structure, localization, and regulatory properties, PP2A subunits represent exciting therapeutic targets to modulate altered adrenergic signaling in cardiovascular disease. In addition, PP2A could affect diabetes mellitus-related cardiomyopathy by regulation of autophagy and apoptosis through ROS dependent pathway^33^. The overall findings highlight the hidden role of *PPP2R2A* in the HF patients. Kaushik et al. revealed the marked different expression of Fos in the hypothalamus of rats with heart failure^34^. However, evidence for a direct link between Fos and HF in humans is still lacking. Another candidate marker gene was *DUSP1* which was up-regulated in the TF-miRNA-mRNA network. It has been believed that mitogen-activated protein kinases (MAPKs) are activated in the heart by disease-inducing and stress-inducing stimuli, where they participate in hypertrophy, remodeling, contractility, and heart failure^35^. Mannix et al. proved that dual-specificity phosphatases (DUSPs) could directly inactivate each of the MAPK terminal effectors, potentially serving a cardioprotective role^35^. In summary, *TMEM87A*, *PPP2R2A* and *DUSP1* have the potential to be biomarkers for HF, and recent studies from several laboratories have provided strong evidence for these three genes.

Recently, the important fractures of miRNA associated with HF have received increasing attention^36^. miRNA-mediated feed-forward loop not only plays an important regulatory role in the growth, differentiation, and metabolism of normal tissues or cells but also participates in the regulation of the pathogenesis of many diseases, such as atherosclerosis, cancer and schizophrenia. The inhibition of miR-92a expression in mouse models with hindlimb ischemia and myocardial cell infarction was found to improve vascular growth and promote the functional repair of damaged tissues significantly^37^. These outcomes were consistent with Angelika’s findings showing that miR-92a may play an important role in heart failure caused by myocardial infarction, during which the expression of SP1 and ROHB was upregulated, and SP1 has been found to regulate ROHB and other signaling molecules^38^. Therefore, we speculated that a network system has been established involving the SP1-miR-92a-ROHB signaling pathway.

The dedifferentiation, proliferation, migration and phenotypic changes of vascular smooth muscle cells in the intima during systolic and quiescent phases play important roles in the occurrence and development of atherosclerosis. Karagiannis et al.^39^ used weakly oxidized low-density lipoprotein in mildly oxidized low-density lipoprotein to induce the dedifferentiation of smooth muscle cells and found that a complex regulatory network regulates the phenotypic transformation of vascular smooth muscle cells. In this complex network, several “linking” genes (including some complex multiple sub-enzyme components involved in the terminal phase of cholesterol synthesis), miRNAs (such as miR-203, miR-511, miR-590-3p, and miR-346/miR-1207-5p/miR-4763-3p), G-protein coupled receptors (GPCR) family (such as GPR1, GPR64, GPRC5A, GPR171, GPR176, GPR32, GPR25 and GPR124) and signal transduction pathways are involved. These genes and miRNAs may play important roles in the pathological process of heart failure. Whether these miRNAs, protein molecules and genes function through some special regulatory mode, such as a feed-forward loop or feedback loop, needs further study and discussion.

In the study, we constructed a network analysis. The strengths of the study are the network analysis among miRNA, TF and target gene, indicating that we have implemented reliable routines for miRNA, TF and target gene analysis. Other strengths of the study are adjudication of all diagnoses among patients with HF or non-HF. However, One limitation still exists in our study. The miRNA-TF-target gene interactions were predicted by GO database, lacking experimental validation. All these results suggested that the occurrence and progression of heart failure may be caused by the interaction of numerous genes, miRNA and TF. Although our study did not further verify these genes or miRNAs in animal experiments, it also provided some reference for future studies related to heart failure. Further experimental study will further verify their interactions.

## Conclusion

*TMEM87A*, *PPP2R2A*, *DUSP1,* and miR-92a have great potential as biomarkers for heart failure. The integrated analysis of mRNA expression spectrum and microRNA-transcription factor-gene revealed the regulatory network of heart failure, which may provide clues to the alternative treatment of heart failure.

### Supplementary Information

Supplementary Information.

## Data Availability

Publicly available datasets were analyzed in this study. This data can be found here: Gene Expression Omnibus: GSE59867, GSE9128, GSE134766. Data is provided within the manuscript or supplementary information files.

## Abbreviations

BP: Biological process
MF: Molecular function
CC: Cellular component
DEGs: Differentially expressed genes
GEO: Gene Expression Omnibus
KEGG: Kyoto encyclopedia of genes and genomes
HF: Heart failure
TF: Transcription factor
PPI: Protein-protein interaction
GPCR: G-protein coupled receptors
FBL: Feedback loop
FFL: Feed-forward loop
